# Analysis of wild macaque stone tools used to crack oil palm nuts

**DOI:** 10.1098/rsos.171904

**Published:** 2018-03-21

**Authors:** T. Proffitt, V. L. Luncz, S. Malaivijitnond, M. Gumert, M. S. Svensson, M. Haslam

**Affiliations:** 1 Institute of Archaeology, University College London, 31–34 Gordon Square, London WC1H 0PY, UK; 2 Institute of Cognitive and Evolutionary Anthropology, University of Oxford, 64 Banbury Road, Oxford OX2 6PN, UK; 3 Department of Biology, Faculty of Science, Chulalongkorn University, Bangkok 10330, Thailand; 4 National Primate Research Centre of Thailand, Chulalongkorn University, Saraburi, Thailand; 5 School of Social Sciences, Nanyang Technological University, Singapore 637332, Singapore; 6 Department of Social Science, Oxford Brookes University, Oxford OX3 0BP, UK; 7 School of Archaeology, University of Oxford, Oxford OX1 2PG, UK

**Keywords:** primate archaeology, percussive technology, human evolution, hammerstone, *Macaca fascicularis*, use-wear

## Abstract

The discovery of oil palm (*Elaeis guineensis*) nut-cracking by wild long-tailed macaques (*Macaca fascicularis*) is significant for the study of non-human primate and hominin percussive behaviour. Up until now, only West African chimpanzees (*Pan troglodytes verus*) and modern human populations were known to use stone hammers to crack open this particular hard-shelled palm nut. The addition of non-habituated, wild macaques increases our comparative dataset of primate lithic percussive behaviour focused on this one plant species. Here, we present an initial description of hammerstones used by macaques to crack oil palm nuts, recovered from active nut-cracking locations on Yao Noi Island, Ao Phang Nga National Park, Thailand. We combine a techno-typological approach with microscopic and macroscopic use-wear analysis of percussive damage to characterize the percussive signature of macaque palm oil nut-cracking tools. These artefacts are characterized by a high degree of battering and crushing on most surfaces, which is visible at both macro and microscopic levels. The degree and extent of this damage is a consequence of a dynamic interplay between a number of factors, including anvil morphology and macaque percussive techniques. Beyond the behavioural importance of these artefacts, macaque nut-cracking represents a new target for primate archaeological investigations, and opens new opportunities for comparisons between tool using primate species and with early hominin percussive behaviour, for which nut-cracking has been frequently inferred.

## 1. Introduction and background

Stone tool percussion is of increasing interest to primatology, archaeology and primate archaeology [1]. The durable nature of lithic artefacts makes them the prime candidate for tracing the development of primate and hominin percussive technologies through time [2,3]. Habitual stone tool use is rare among non-human primates (hereafter, primates), and to date only three species are known to use stones to access encased food in the wild: West African chimpanzees (*Pan troglodytes verus*) [4,5], bearded capuchins (*Sapajus libidinosus*) in Brazil [6,7] and long-tailed macaques (*Macaca fascicularis*) in Thailand and Myanmar [8].

Following a largely overlooked nineteenth-century report [9], long-tailed macaque stone tool use was rediscovered a decade ago on islands off the Thailand coast [10]. These monkeys use stone tools in a range of foraging and food-processing activities, primarily within the intertidal zone. The most intensively studied populations, on Piak Nam Yai (PNY) Island in Laem Son National Park and Koram Island at Khao Sam Roi Yot National Park, use stone tools in two primary manners, termed axe hammers and pound hammers [11–15]. Axe hammers are generally used to open sessile prey such as oysters, and develop distinctive wear patterns on their narrower points, while pound hammers are used to open various gastropods and sea almonds (*Terminalia catappa*) [16]. The percussive damage associated with pound hammers is generally located on the larger, flatter surfaces of the hammerstone, allowing differentiation of macaque axe and pound hammers in both observational [17] and archaeological [12,13] contexts.

Recently, macaques on Yao Noi Island (YNI) in Ao Phang Nga National Park, Thailand, were observed using stone tools to process oil palm (*Elaeis guineensis*) nuts away from the intertidal zone, in the evergreen rainforest [14] (electronic supplementary material, S1). Oil palm trees were first introduced to the island by humans in the past few decades. Both wild chimpanzees and capuchins target nuts as part of their lithic tool behaviour, with chimpanzees processing species such as *Coula edulis* and *Panda oleosa* in the Taï forest and *E. guineensis* in Bossou in West Africa [18–20]. Bearded capuchins crack local palm nuts (e.g. *Astrocaryum campestre* and *Orbignya* sp.) and cashew nuts (*Anacardium* sp.) in semi-arid northeastern Brazil [6,21,22].

Chimpanzee oil palm processing in Bossou, Guinea, is one of the more comprehensively studied stone behaviours, with reports of social learning [23], tool movement and selection [20,24,25] and use-wear on stone hammers [26]. Oil palm nuts contribute a significant percentage of the food consumed by the chimpanzees at Bossou, and are used throughout the year. Their consumption, however, peaks when another fruit becomes scarce [27]. The discovery of stone tool-mediated processing of oil palm by macaques therefore provides an opportunity to examine primate percussive technology both within and between species.

Percussive technology forms an important component of the earliest hominin technological and behavioural repertoires. Evidence of repeated percussive activity is clear on the earliest known tools: large cores and passive hammers from Lomewki 3, West Turkana [28,29]. Although this damage may be associated with bipolar knapping rather than food processing, it demonstrates the antiquity of percussive behaviour in the palaeoanthropological record. Percussive activities are also evident within the Oldowan [30–33], with both passive (anvil) and active (hammerstone) elements identified in a range of raw materials. Hammerstones were commonly used for knapping, although anvils were probably used for a range of activities, including bipolar reduction [34], nut-cracking [35] and bone-breaking [30]. As the percussive behaviour of extinct hominins is not directly observable, the study of extant non-human primates' percussive activities is an increasingly valuable source for interpreting the archaeological record.

Here, we present the first comprehensive technological and use-wear characterization of Old World monkey oil palm processing hammerstones. We describe the stone tools, along with associated percussive damage, to facilitate future identification and comparison of such artefacts in the primate and hominin archaeological records.

## 2. Material and methods

**Figure 1.**
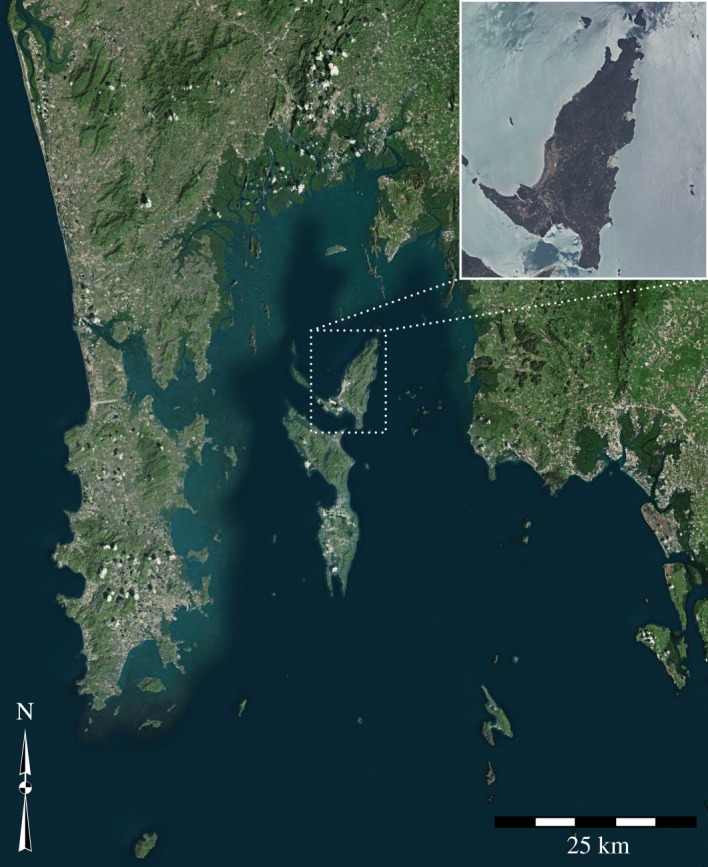
Map of YNI within the Ao Phang-Nga National Park, Thailand (adapted from [14]).

Macaque hammerstones were collected from YNI in October 2016. The study site is located in a coastal forest in the north of YNI (8°10.838′ N, 98°37.746′ E) (figure 1), and detailed descriptions of the environment of oil palm nut-cracking locations, along with the associated behaviour, are published elsewhere [14]. Hammerstones were identified by their association with large stone anvils on which macaques had exclusively cracked oil palm nuts [14], and were selected based on clear macroscopic percussive damage. A sample of these hammerstones (*n* = 13; the ‘use-wear sample’) (electronic supplementary material, S2) was collected from a number of anvil sites and analysed using a techno-typological, macro and microscopic approach. In addition, data on maximum dimensions and weight were collected in the field for a larger sample of hammerstones (*n* = 335; the ‘transect sample’) (electronic supplementary material, S2) identified at anvils on 13 line transects placed throughout the study area [14]. Dimensional measurements for the transect sample were recorded to the nearest 5 mm, with weight being measured to the nearest gram.

### 2.1. Technological analysis

In recent years, a combination of techno-typological and microscopic use-wear analyses have been applied to hominin [35,36], ape [26,37] and experimental [34,38] percussive stone technologies. These methods have produced a growing dataset of artefacts and damage associated with a wide range of percussive activities, and we follow the same methods here. All hammerstones were classified following protocols set out by Mora & de la Torre [39] for percussive technology and Proffitt *et al*. [40] for classification of primate percussive hammerstones. Attributes documented for each hammerstone included the morphological shape of the original cobble, a qualitative assessment of the degree of percussive damage and its spatial distribution, the number of surfaces that possess percussive damage, and the morphology of the area where percussive damage was identified. We also documented the number and dimensions of any flake scars, and percentage of cortex coverage.

### 2.2. Microscopic use-wear analysis

Microscopic analysis was conducted on all use-wear sample hammerstones using a low magnification (less than 100×) Leica S9APO stereo microscope equipped with 1–8× objective lenses and a 10× eyepiece, and photographs were taken using a 3.1 Megapixel EC3 digital microscope camera attached to the microscope. Microscopic use-wear was characterized following standardized methods and terminology for percussive and ground stone artefacts [41], which have been applied successfully to both hominin [35] and primate [42,43] percussive lithic material.

### 2.3. Macroscopic use-wear analysis

Macroscopic use-wear followed the procedure for macaque stone tools described in Haslam *et al*. [17], which calculates a use action index (UAI) score for each tool. This procedure evaluates the extent and intensity of macroscopically visible crushing, pitting and fracture damage to each of 10 tool zones, with five zones on each main face of the tool. The UAI was developed by comparing the crushing damage on the narrow end of a tool to that on the broad faces, in order to distinguish macaque axe and pound hammers. It was designed specifically to allow rapid recording and analysis of use-wear in the field, to avoid the need for specialist equipment or removal of tools from a field site, where this might disrupt natural animal behaviour. The UAI is calculated as: UAI = 1.21 + (1.07Cp)−(1.22Cf)−(0.014Wt), where Cp is crushing on the tool's narrow end, Cf the crushing on the tool faces, and Wt the tool's weight in grams. A negative result indicates a pound hammer (more wear on the faces) and a positive result suggests an axe hammer (more wear on the tool end), with over 90% reliability [17]. In addition to the UAI, wear intensity scores for the individual zones give an indication of wear development on tool ends, faces or edges, which can be compared separately between different tool assemblages. In this study, all assessments of similarity between assemblages used a two-tailed Mann–Whitney *U*-test (*α* = 0.05).

### 2.4. Intra- and inter-species comparisons

To facilitate intra-species comparison of hammerstone use, data on tools used by macaques for processing sessile oysters (axe hammers), and marine gastropods (pound hammers) were taken from [17], while data for sea almond hammerstones (also classified as pound hammers) were taken from Falótico *et al*. [16]. Dimensional comparisons used the combined data of oil palm hammerstones derived from both transect and use-wear samples. To identify statistical similarities and differences between these datasets, we performed one-way analysis of variance (ANOVA) and Tukey post hoc (*α* = 0.05) tests.

Inter-species comparison of oil palm nut processing hammerstones included published data for chimpanzees from natural (non-experimental) nut-cracking sites at Bossou. The majority of available data on Bossou chimpanzee stone tools is associated with an open air experimental laboratory, where locally sourced raw material is selected and provided to the chimpanzees by humans, creating an artificial assemblage [20]. The artificial selection of the hammerstones used at the Bossou experimental site precludes a direct comparison of hammerstone dimensions with wild species, as they do not necessarily reflect a naturalistic setting [44]. To overcome this issue, only data on oil palm hammerstones identified from contexts in the wild were used. Dimensional data were derived from two separate wild studies of the same field site, Sakura & Matsuzawa [25] (Bossou 1), and Humle & Matsuzawa [45] (Bossou 2). As full datasets were not published in these reports, all comparisons were conducted on mean values and standard deviations using a Welch unpaired *t*-test.

## 3. Results

### 3.1. Techno-typological analysis

All 13 macaque nut-cracking hammerstones from the use-wear sample are considered to be active elements using recent definitions [31,32,39]. They can be subdivided into complete hammerstones (*n* = 5, 38.5%), flaked hammerstones that possess one or more small and non-invasive unintentional flake detachments (*n* = 6, 46.2%), and hammerstones with fracture angles (*sensu* [31]) (*n* = 2, 15.4%). The hammerstones possess a mean length, width and thickness of 110 × 89 × 56 mm, and an average weight of 751 g (table 1). The weight shows a relatively high degree of variability (142–2067 g), with a standard deviation of over half a kilogram (518 g). The use-wear sample hammerstones are rounded or weathered limestone cobbles, which possess a fine-grained and homogeneous internal structure. Most of these cobbles are roughly tabular (*n* = 7, 53.8%) in morphology; however, both plano-convex (*n* = 3, 23.1%) and irregular (*n* = 3, 23.1%) cobbles were also used. Based on the larger transect sample, limestone is the most commonly used material at the site (*n* = 252, 74.6%), followed by laterite (*n* = 73, 21.6%), and granite (*n* = 13, 3.8%).

**Table 1.** Dimensions of macaque oil palm nut-processing hammerstones, YNI, Thailand.

artefact ID	length (mm)	width (mm)	thickness (mm)	weight (g)
*use-wear sample* (*n* = *13*)				
100	123.3	90.1	49.9	678.8
101	70.4	58.0	29.8	142.0
102	108.7	93.5	40.1	525.5
103	165.7	121.7	95.7	2067.3
104	104.6	101.0	54.9	746.0
105	111.7	106.9	57.7	752.4
106	125.1	104.1	57.6	880.9
107	123.4	92.4	84.6	1457.4
108	85.7	81.1	48.4	431.1
109	112.3	79.4	60.7	509.1
110	78.8	67.4	53.7	248.6
111	93.7	66.8	48.2	418.4
112	130.6	96.5	50.1	909.8
minimum	70.4	58.0	29.8	142.0
maximum	165.7	121.7	95.7	2067.3
mean	110.3	89.1	56.3	751.3
standard deviation	25.1	18.1	17.2	518.4
*transect sample* (*n* = *335*)				
minimum	40	30	20	59
maximum	220	650	660	5000
mean	90.7	64.7	50.4	412.2
standard deviation	27.2	38	57.5	591.3
*combined hammerstone dataset* (*n* = *348*)				
minimum	40	30	20	59
maximum	220	650	660	5000
mean	91.4	65.7	50.6	424.8
standard deviation	27.3	27.3	37.7	591.6

The majority of the hammerstones possess a substantial degree of macroscopically visible percussive damage. This damage is primarily present on all six planes (*n* = 10, 76.9%), with only a single example each of damage located on three (7.7%), four (7.7%) and five (7.7%) planes. Damage is typically clustered on a combination of both the flat surfaces and either along ridges or on raised convex surfaces, as well as in some cases being located on a combination of flat, convex and ridged regions of the hammerstone.

**Figure 2.**
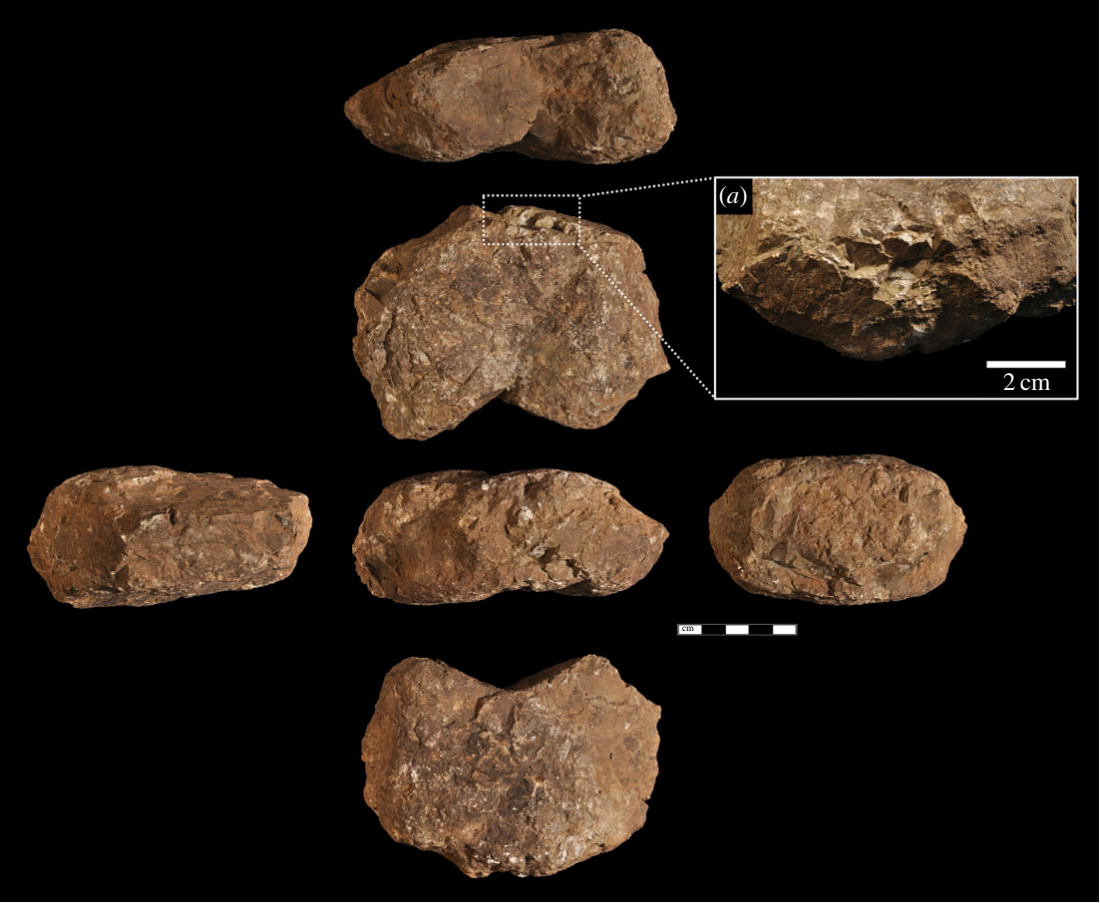
Macaque oil palm hammerstone made of fine-grained limestone, from YNI, Thailand (scale 5** **cm). Detail (*a*) of clustered angular detachments due to repeated mis-hits.

**Figure 3.**
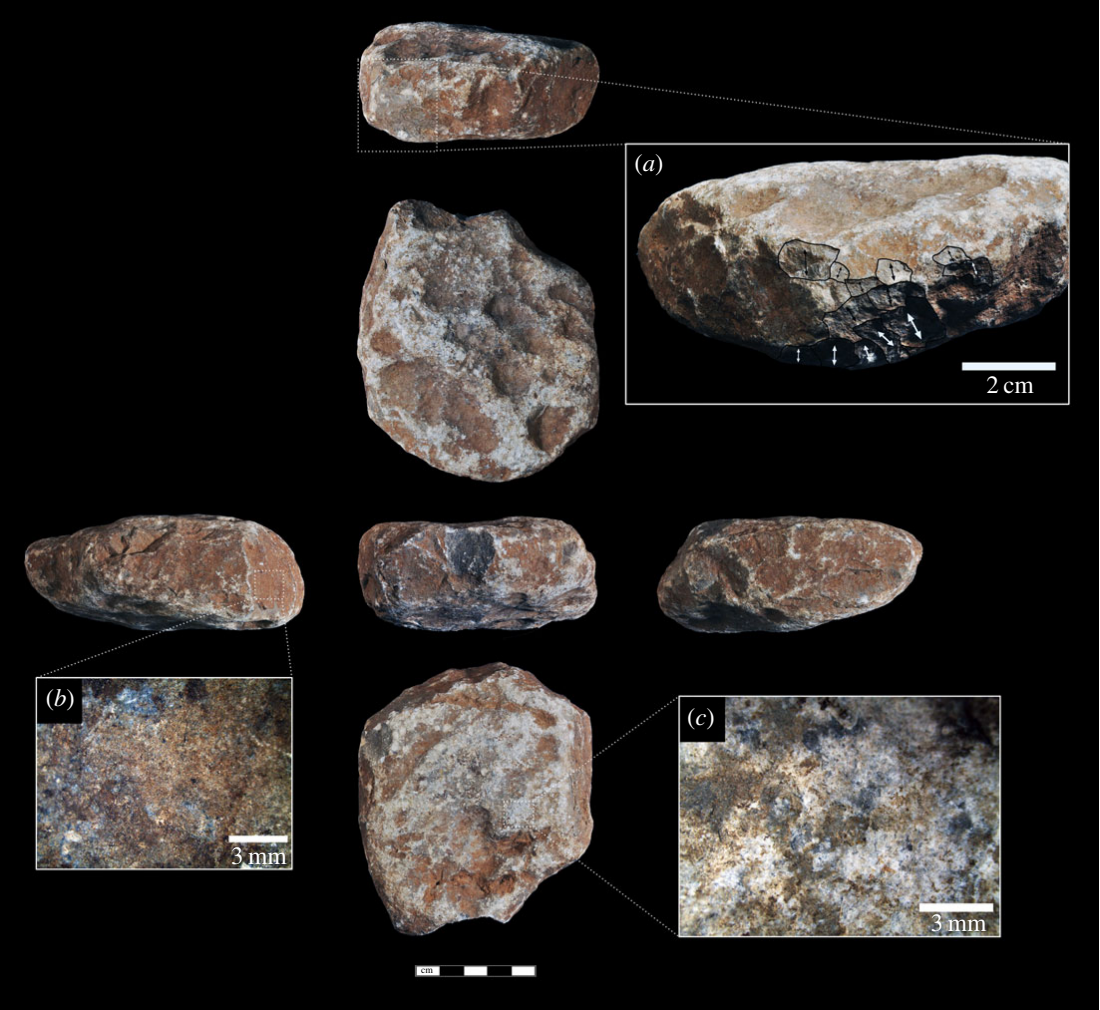
Heavily battered macaque oil palm hammerstone (scale 5** **cm). Details show (*a*) clusters of angular detachments along intersecting ridges due to mis-hits and (*b,c*) microscopic crushing of the working surfaces and clusters of angular detachments along intersecting ridges due to mis-hits. Arrows indicate the direction of the detachments.

Macaque hammerstone use has resulted in instances of one or more flake removals along intersecting planes. Six of the hammerstones have these detachments, with an average of three removals (range = 1–6). These detachments are small and non-invasive, on average measuring 15 × 10 mm in maximum dimension. The removals either occur along naturally acute angles, resulting in small conchoidal removals, or are detached from natural oblique intersections and can be considered typical hammerstone detachments (*sensu* [46]). They most likely occurred as a result of mis-hits or oblique strikes on a nut, resulting in forceful contact between the hammerstone and a stationary stone anvil [43]. Two hammerstones possess percussive damage that can be considered similar to hammerstones with fracture angles [32,39]. Although not as developed as some archaeological examples, the hammerstones with fracture angles produced during macaque nut-cracking possess a series of simultaneous, angular, step-terminating removals located on either side of an intersecting obtuse ridge between two planes. In both cases, the damage is localized rather than pervasive. In some cases, this intersection can take on a partly bifacial morphology (although not bifacially flaked), as a consequence of continued accidental percussive contact along the ridge (figures 2 and 3).

### 3.2. Macroscopic use-wear analysis

At a macroscopic level, the percussive use-wear on the hammerstones is clear, and typically presents as discrete discoloured regions with a pitted and crushed morphology. The results of the UAI analysis are presented in table 2 (electronic supplementary material, S2). All hammerstones returned a UAI result indicative of primary use of the broad tool faces to pound hard objects, confirming the applicability of the UAI method, which was developed using macaque gastropod pounding tools, to wild macaque oil palm nut-cracking.

**Table 2.** UAI for macaque oil palm nut-processing hammerstones, YNI, Thailand.

artefact ID	UAI	use-action	artefact ID	UAI	use-action
100	−12.1	pounding	107	−22.2	pounding
101	−2.7	pounding	108	−6.8	pounding
102	−9.2	pounding	109	−11.1	pounding
103	−29.1	pounding	110	−5.0	pounding
104	−12.3	pounding	111	−8.6	pounding
105	−11.0	pounding	112	−12.4	pounding
106	−14.2	pounding			

### 3.3. Microscopic use-wear analysis

**Figure 4.**
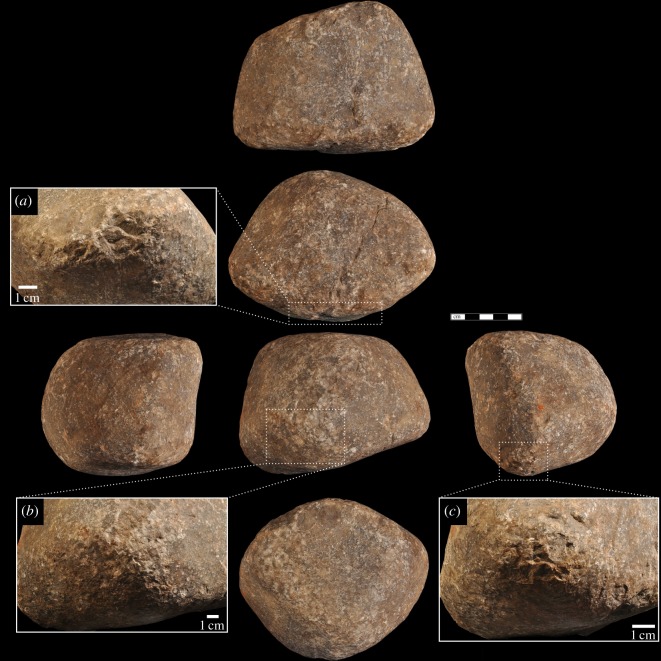
Heavily battered macaque oil palm hammerstone from YNI, Thailand (scale 5** **cm). Details (*a–c*) show heavy pitting and crushing on the rounded extremities of the hammerstone and clusters of angular detachments along intersecting ridges.

At a microscopic level, macaque hammerstone percussive damage can be separated into two main categories: (i) crushing and (ii) polish or abrasion (table 3). The crushed regions have multiple, superimposed impacts located broadly across entire surfaces, but most intensely on intersecting ridges and convex surfaces (figure 3). In other words, the majority of the crushing damage is located on topographically raised regions of the hammerstones. The superimposed impact points have, over time, caused significant modification to the surface of the stone. In most of the cases, this modification results in a flattening of the ridges and raised convex regions; however, in some cases, the repeated impacts have pitted the stone, resembling pecking modification (figure 4).

**Table 3.** Microscopic use-wear on macaque oil palm nut-processing hammerstones, YNI, Thailand.

artefact ID	microscopic analysis	impact points	crushing	micro- fractures	step fractures	abrasion/ polish	striations in polished regions	striations in non-polished regions
100	yes	yes	no	yes	yes	no	no	no
101	yes	yes	yes	no	no	no	no	no
102	yes	yes	yes	no	yes	no	no	yes
103	yes	yes	yes	yes	yes	yes	no	no
104	yes	yes	yes	no	no	yes	yes	no
105	yes	yes	yes	yes	no	yes	yes	yes
106	yes	yes	yes	yes	yes	no	no	no
107	yes	yes	yes	yes	no	no	no	no
108	yes	yes	yes	no	no	yes	yes	no
109	yes	yes	yes	no	no	yes	yes	no
110	yes	yes	no	yes	no	no	no	no
111	yes	yes	no	no	yes	no	no	no
112	yes	yes	yes	no	yes	yes	yes	no

Where the repeated superimposed impact points are located close to an intersecting edge, they are associated with small step-terminating fractures and micro-fractures. Similarly, if located away from the edge in a more interior position, these areas of high-intensity percussive damage are often associated with micro-fractures. These micro-fractures radiate from single or multiple percussive impacts and are detached perpendicular to the direction of the impact, resembling a type of surface peeling. In addition to the crushed regions and micro-fractures, a number of hammerstones possess linear wear marks that are sparsely located across the surface. These abrasions occur individually and do not possess a consistent directionality. The presence of such abrasions suggests a degree of horizontal movement during percussive strikes, when the hammerstone comes into contact with the anvil.

**Figure 5.**
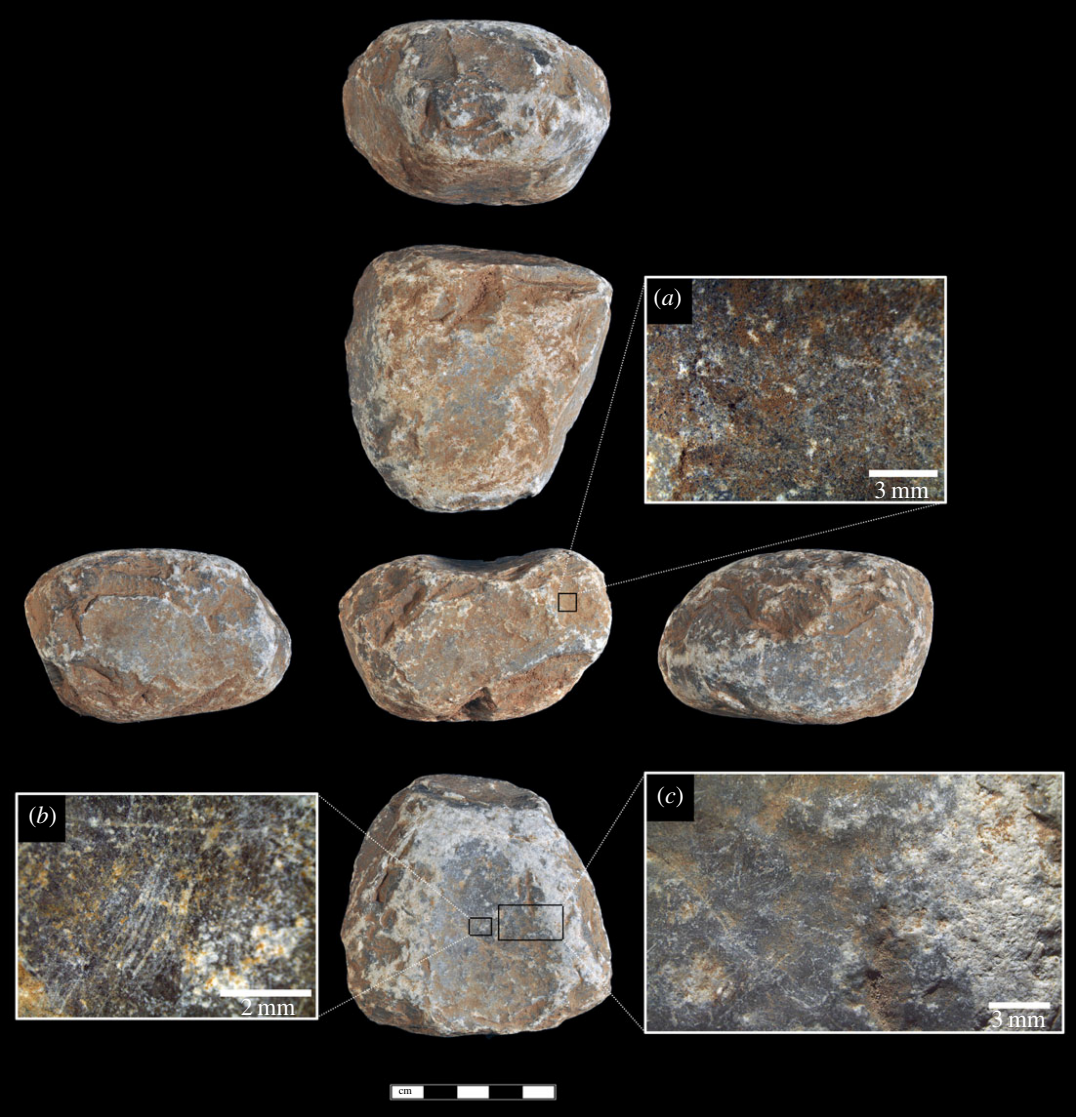
Heavily battered macaque oil palm hammerstone from YNI, Thailand (scale 5 cm). Details show (*a*) repeated impact points on the working surface of the hammerstone, and (*b,c*) heavily crushed regions immediately adjacent to areas of striations and polish.

The intensive, high-energy percussive damage characterized above is primarily located along the raised regions of the hammerstones, and is a result of an interaction between the hammerstones and the anvil stone. However, percussive damage is also present at a micro level within central concave regions of a smaller number of the use-wear sample hammerstones (*n* = 6, 46.1%), caused by contact with the oil palm nut. This use-wear is characterized by restricted regions of polish and abrasion located towards central natural concavities of the main horizontal planes. This wear creates a smooth, slightly polished surface, clearly differentiated from the surrounding intensive percussive damage (figure 5). The polish develops through the initial abrasion of topographic high points within the central depression, including multiple micro-striations resulting from contact with palm nut husks and fine sediment particles attached to the nut surface. These micro-striations often occur in clusters that share the same directionality; however, at a broader scale, the clusters show no clear directionality within the polished areas.

### 3.4. Intra- and inter-species comparisons

**Figure 6.**
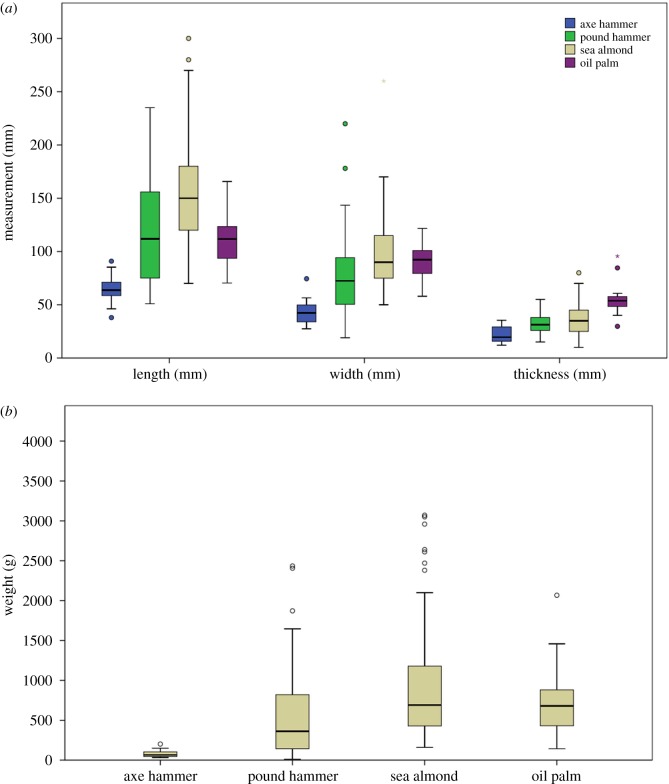
Dimensions of macaque oil palm hammerstones compared to sessile oyster tools (axe hammers), marine gastropod tools (pound hammers) and sea almond tools (pound hammers). (*a*) Box plot of length (mm), width (mm) and thickness (mm) for all hammerstones. (*b*) Box plot of weight (g) for all hammerstones.

Comparing wild macaque percussive stone tools across processed foods, oil palm hammerstone sizes fall at the lower end of the range. However, there is also substantial overlap in terms of dimensions with all other macaque percussive tasks (figure 6). A one-way ANOVA test indicates a significant difference in all dimensions (length (*F*_3,506_ = 108.494, *p* < 0.001), width (*F*_3,506_ = 27.127, *p* < 0.001), thickness (*F*_3,506_ = 5.348, *p* = 0.001)) and weight (*F*_3,506_ = 21.056, *p* < 0.001) between hammerstones used for processing oil palm nuts, sea almonds, gastropods and oysters. A Tukey post hoc test shows that macaque pound hammers used to open oil palm nuts are significantly lighter (weight *p* < 0.001), shorter (length *p* < 0.001) and narrower (width *p* < 0.001), but also significantly thicker (thickness *p* = 0.029) than those used to crack sea almonds. Oil palm hammerstones are also significantly longer (length *p* = 0.006) than oyster axe hammerstones and significantly shorter (length *p* < 0.001) than pound hammers used to process gastropods. There is no significant difference in weight, width and thickness between oil palm hammerstones and those used to open either oysters or gastropods.

Comparing the UAI values between macaque hammerstone assemblages, the YNI oil palm hammerstones were not significantly different from stone tools used by wild macaques for either gastropod processing (*n* = 42, *U* = 187, *z* = 1.69, *p* = 0.09) or sea almond processing (*n* = 97, *U* = 661.5, *z* = −0.28, *p* = 0.78) on PNY in Laem Son National Park [16,17]. There is also no significant difference when comparing only pitting wear on the broad surfaces between the oil palm hammerstones and PNY gastropod pound hammers (*U* = 233.5, *z* = 0.77, *p* = 0.44). However, there is significantly more intense crushing wear on the faces (*U* = 476, *z* = −4.01, *p* < 0.001), edges (*U* = 546, *z* = −5.4, *p* < 0.001) and narrower ends (*U* = 505, *z* = −4.59, *p* < 0.001) of the oil palm nut-cracking hammerstones than on the PNY gastropod processing pound hammers. There is also more intense fracture damage on the narrower ends of the oil palm nut-cracking hammerstones than for the same position on the PNY gastropod processing pound hammers (*U* = 391, *z* = −2.33, *p* = 0.02). There is no difference in fracture intensity on the edges of these two tool assemblages. Finally, comparing wear on the faces of macaque stone tools used to process encased plant foods—oil palm and sea almond—there is significantly more pitting on the oil palm hammerstones (*U* = 859.5, *z* = −2.12, *p* = 0.034).

**Figure 7.**
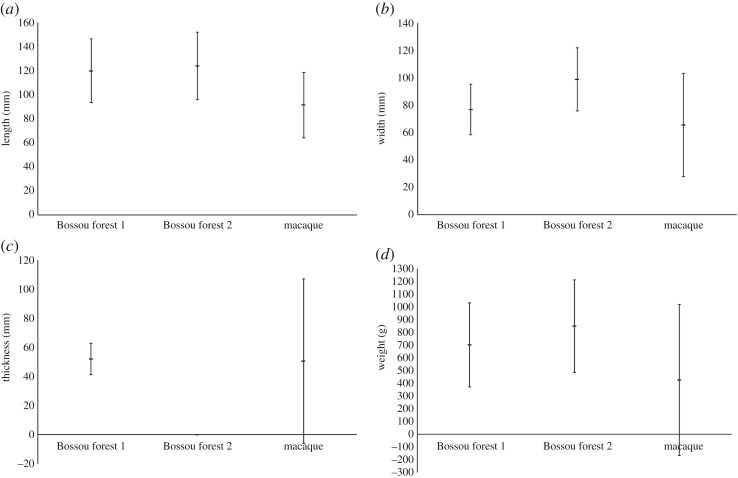
Mean dimensions ((*a*) length, (*b*) width, (*c*) thickness, (*d*) weight) and standard deviations of YNI macaque and Bossou chimpanzee oil palm processing hammerstones.

The macaque oil palm hammerstones vary significantly from those used in natural settings by wild Bossou chimpanzees (Bossou 1: Sakura & Matsuzawa [25], and Bossou 2: Humle & Matsuzawa [45]). There are significant differences in length, width and weight, with all three being smaller for the macaque hammerstones, with no significant difference in thickness (figure 7 and table 4). However, there is considerable overlap in hammerstone dimensions, with several macaque hammerstones falling within the size range used by Bossou chimpanzees.

**Table 4.** Inter-species comparison of oil palm hammerstone dimensions from wild macaques on YNI and chimpanzees at Bossou, using Welch unpaired *t*-test (*α* = 0.05).

	length (mm)			width (mm)			thickness (mm)			weight (g)		
	site	mean	s.d.		mean	s.d.		mean	s.d.		mean	s.d.
Bossou 1 *n* = 35	120	26.5		77	18.6		52	10.8		700	330	
Bossou 2 *n *= 109	124	28		99	23		—	—		848.6	363.4	
YNI (this study) *n* = 348	91.4	27.3		65.7	37.7		50.6	56.5		424.8	591.6	

*comparison*	*t*	d.f.	*p*-value	*t*	d.f.	*p*-value	*t*	d.f.	*p*-value	*t*	d.f.	*p*-value
YNI versus Bossou 1	6.07	41	0.0001	3.02	66	0.0036	0.40	274	0.6925	4.29	58	0.0001
YNI versus Bossou 2	10.67	177	0.001	11.14	300	0.0001	—	—	—	9.00	297	0.0001

## 4. Discussion

The identification of this new wild macaque nut-cracking behaviour and associated stone tools [14] has wide relevance. First, it allows us to compare variation between tool types and activities in the non-human primate and hominin archaeological records, assisting in the development of new hypotheses on tool selection, production and use. Second, the fact that members of multiple wild primate species (macaques and chimpanzees) habitually use stone tools to process the same food source, oil palm nuts, allows us to directly compare the form and damage patterns of their tools during use. Finally, it offers an opportunity to examine the degree of skill employed by each species in the same task, and to assess inter-species differences in tool selection and mode of use.

Tool-use variation within a single primate species provides insights into the range of behaviour potentially identifiable in the primate archaeological record. For example, the fact that wild macaques displayed a tool-use-mediated, flexible response to rapidly changing environments [14] to take advantage of the new oil palm food source has implications for identifying changes in their past behaviour. Macaque stone hammers show a significant and identifiable variation in percussive damage location and intensity in relation to the specific task being performed [17], allowing us to reconstruct their behaviour from the archaeological record [12,13]. In addition, lithic hammers used by macaques to process different food types, such as sea almonds and shellfish, show significant size and use-wear differences [16]. Each of these characteristics offers a target for interpreting stone tools used by macaque ancestors. The recent onset of this behaviour within the YNI macaques [14] enables the archaeological documentation of the development of this behaviour within a single wild primate community. Furthermore, studying variation within a single species' hammerstone use helps lay the foundations for understanding the archaeological record of other stone-tool using hominins and non-human animals.

The percussive damage on macaque oil palm processing hammerstones differs from that seen on their other tools. For example, there is substantial damage across all planes of the tool, with the majority located on the flatter horizontal planes, in contrast with the end-damage seen on macaque oyster processing tools. Unlike on typical gastropod hammerstones used at the coast, crushing damage on oil palm tools used in inland forests is not consistently confined to the central region of the working surface; instead, it is located across topographic high regions and intersecting ridges, caused by repeated miss-hits of the hammer against the stone anvil. This may be a consequence of differing anvil morphologies at the different ecological settings, with oil palm anvils often possessing an undulating morphology (electronic supplementary material, S1). As such, they are more likely to come into contact with the hammerstone, compared to the tide-worn flatter surface of coastal anvils. Our data also indicate that oil palm processing does not produce the same degree of concentrated damage in the centre of the working surface seen in gastropod pound hammers (although field observations noted a degree of small pit formation during use on some hammerstones). Instead, oil palm damage is characterized by a slight polish and abrasion of the striking surface.

The discovery of macaque oil palm nut processing provides the first opportunity to compare the artefactual signature of two non-human primates known to engage in the same behaviour with the same nut species. Although chimpanzee stone tool use at Bossou has been known for decades, there are no published reports providing detailed technological and use-damage attributes of tools used outside the field laboratory. From the few available studies that include average tool sizes at natural settings in Bossou, however, our analysis shows that hammerstones used by chimpanzees for cracking *E. guineensis* are significantly larger than those of the YNI macaques. This finding may indicate that both species are selecting hammerstones based in part on their body size and strength, instead of converging on an optimum hammerstone weight and size for the efficient processing of oil palm nuts. The macaque hammerstone transect sample shows a prevalence of limestone tools, followed by those of laterite and granite. This hammerstone selection may mirror the natural ratios of these raw materials in the YNI landscape, although future work will be required to quantify that comparison. However, all granite stones that were identified in transect surveys had been used as hammerstones, which may indicate a degree of raw material selectivity [14]. At Bossou, the chimpanzees also seem to preferentially select specific raw materials for oil palm nut-cracking in natural settings. For example, at the Moblim SA13 site, locally available granite was preferentially selected over other raw materials [20].

No use-wear studies are currently available for stones used by Bossou chimpanzees away from the on-site field laboratory. However, with the caveat that differences may exist between natural behaviour and that found at the laboratory, we can use a recent study of experimental stones from Bossou [26] to make an initial comparison of chimpanzee damage patterns with those of the YNI macaques. Many of the chimpanzee oil palm processing stone tools possessed central pits, some of which were surrounded by slightly polished regions, located on the topographic high regions. The degree of pitting on those tools was largely dictated by the quality of the raw material, with much of it associated with percussive damage on a friable oxide outer coating. By contrast, none of the macaque tools studied here had artificially produced central pitting, even if naturally occurring concavities were present on the tool surface. We note, however, that this finding will need to be tested against other used raw materials at YNI, particularly those that are more friable than limestone.

Unlike the Bossou chimpanzee hammers, percussive damage identified on the YNI macaque tools is typically located along topographic high regions and intersecting ridges, and consists mainly of high-energy percussive damage, including impact points and crushing. Although impact points were identified on the chimpanzee experimental tools, they were primarily located around the peripheral edges. Polished regions on active chimpanzee hammers were located along pronounced topographic ridges, although they were not substantial. Polish on the macaque hammers develops, not around the outside of concavities, but inside, and on regions in direct contact with the oil palm nut. In addition, on macaque tools, the polish is associated with numerous minute striations.

Some of the oil palm hammerstones at Bossou occasionally fracture; however, those reported are relatively large detachments, big enough occasionally to be used themselves as hammerstones and other tools [20,24]. In the experimental Bossou study, direct percussive damage was largely confined to the two opposed horizontal planes [26], with very little mechanical alteration to the intersecting ridges between planes, other than the occasional impact point. Macaque hammers have damage located on all planes. The differences in percussive damage location and intensity on the tools of the two primates may be due to difference in raw material types, with the raw material from Bossou being friable and susceptible to mechanical pitting [26]. On the other hand, the difference in the degree and location of percussive damage may also suggest a difference in the manual handling of the hammer during use. The presence of large striations on the macaque hammerstones may also indicate that these tools were handled and used with less precision than those in the Bossou experimental study, with the damage resulting from frequent mis-hits against the anvil [43].

Our intra- and inter-species analysis of non-human primate hammerstones highlights the dynamic nature of the development of damage on percussive stone tools. It is clear that a range of inter-linked factors affect the initial development, the location, the extent and morphology of the percussive damage. These include a dynamic relationship between hammerstone raw material, the hardness of the targeted object, the morphology and hardness of the anvil, as well as the degree of dexterity and manipulative ability of the stone tool user and the number of nut-cracking sessions performed by multiple individuals. Disentangling the relative contributions of each of these factors to percussive wear is difficult with non-habituated animals, but the long-term study of habituated wild capuchins shows that collecting such data is not impossible [22,47,48].

The highly battered convex surfaces of the macaque hammerstones resemble, morphologically, typical hominin stone knapping percussive damage. However, this damage is broadly located across all surfaces of the macaque tools rather than confined to a single convex surface as is observed for hominin knapping hammerstones. Nevertheless, hammerstones with fracture angles have been identified in a number of Oldowan assemblages [31,32,39]. These artefacts are described either as hammerstones or active percussive objects, and they exhibit a significant degree of damage along intersecting ridges. They possess extensive, angular percussive damage along one or more intersecting ridges, owing to repeated use. These hammerstones would not have been conducive for knapping activities, as the development of angular fractures prohibits the precise application of force required to initiate conchoidal fracture. Instead, this artefact type may have been used to extract marrow from large bones, or for chopping wood [31].

Although limited to three examples in our macaque use-wear sample, we have found that these wild monkeys reproduce the initial stages of hammerstone angular fracture. On the macaque tools, this damage is confined to multiple impacts on intersecting ridges, resulting in the spontaneous detachment of step-terminating angular fragments along the ridge. This pattern is caused by the hammerstone striking the stone anvil and not the target nut, which instead develops wear towards the centre of the hammerstone surfaces. Our findings therefore provide a further model for the potential behavioural route by which similar artefacts may have been produced by tool-using hominins. We can plausibly hypothesize that a similar, albeit more extensive, process of glancing blows of the hammerstone against the stone anvil may underlie the production of some of the known hominin hammerstones with fracture angles. This hypothesis will, however, require further experimental replication using the same raw materials as those identified in the archaeological record.

## 5. Conclusion

Discoveries of new forms of stone-tool-assisted activity in non-human primates are rare, and as such, they require detailed documentation. Here, we have presented the first technological and use-wear analysis of stone tools used by wild macaques to process oil palm nuts. This artefact type is characterized by a high degree of battering and crushing on most surfaces, which is visible at both macro and microscopic levels. In addition, at a microscopic level, these tools are characterized by varying degrees of polish and striations caused by the repeated interaction with the hard outer surface of the oil palm nut as well as possible adhering particles.

At an intra-species level, these artefacts are significantly smaller on average than those used by wild macaques to process another encased plant food, sea almonds. In general, oil palm hammers fall within the lower average size range of macaque percussive technology. At an inter-species level, macaque oil palm hammerstones are also significantly smaller than those used by wild chimpanzees for processing the same nut. Use-wear is qualitatively different between the tools used by these two species, with the caveats that raw material and context (natural setting versus experimental field laboratory for the chimpanzees) may affect the development of macro and microscopic wear patterns. Macaque oil palm processing joins other forms of primate tool use in assisting with hypothesis formation on the use of hominin percussive artefacts, and highlights the importance of continued field study of non-human primate stone tools using modern archaeological techniques and perspectives.

## Supplementary Material

Supplementary Material 2

## Data accessibility

The datasets supporting this article have been uploaded as part of the electronic supplementary material. These data are deposited at Dryad: https://doi.org/10.5061/dryad.2f5rq [49].
